# The influence of social support on healthcare service use following transport-related musculoskeletal injury

**DOI:** 10.1186/s12913-016-1582-4

**Published:** 2016-07-27

**Authors:** Khic-Houy Prang, Janneke Berecki-Gisolf, Sharon Newnam

**Affiliations:** Monash University Accident Research Centre, Monash University, Building 70, Clayton Campus, Wellington Road, Clayton, VIC 3800 Australia

**Keywords:** Social support, Healthcare service use, Musculoskeletal injury

## Abstract

**Background:**

Social support has been identified as a significant factor in the recovery of individuals with musculoskeletal injury (MSI). However, relatively limited research has examined the mechanisms through which social support influences healthcare service use. This research examines the direct effects, mediating effects and effect modification of social support on healthcare service use among people with MSI sustained in a transport accident.

**Methods:**

The study design was secondary data analysis of cross-sectional surveys of compensated transport accident victims in Victoria in 2010 and 2011, linked to compensation claims and payment records. Analyses included (i) zero-inflated negative binomial and logistic regressions to model healthcare service use (direct effect), (ii) the Karlson, Holme and Breen (KHB) method to assess social support as a mediator of predisposing factors, need factors and healthcare service use (mediation effect), and (iii) interactions to assess social support as a modifier between predisposing factors, need factors and healthcare service use (effect modification).

**Results:**

Results of the direct analyses showed that support from family was associated with lower uptake of allied healthcare services (odds ratio (OR) 2.17; 95 % confidence intervals (CI) 1.21–3.91). Support from friends was associated with lower uptake (OR 1.87; 95 % CI 1.09–3.21) and lower rate (i.e. number of services per person) of allied healthcare services (incidence rate ratio (IRR) 0.65; 95 % CI 0.52–0.83). Support from friends (OR 0.60; 95 % CI 0.38–0.95) was also associated with lower uptake of mental healthcare services. No statistically significant mediation effects were identified for family or friends’ support on the uptake of allied and mental healthcare services. Family support was found to modify the association between socio-economic indexes for areas and mental healthcare service use. In the group that reported having no social support, mental healthcare service uptake in the socioeconomically advantaged group was lower than in the disadvantaged group (OR 0.36; 95 % CI 0.16–0.83).

**Conclusions:**

The findings suggest that social support has a direct and modifying effect on healthcare service use but does not mediate the association between predisposing factors, need factors and healthcare service use. The study findings have implications for the role of social support in the prevention, treatment and intervention of individuals with MSI.

## Background

Musculoskeletal injuries (MSI) are a major public health problem worldwide, contributing to a large burden of disability. According to the World Health Organisation’s Global Burden of Disease study, the majority of admissions for various non-fatal injuries as a result of a road traffic accident were MSI, with almost 50 % of these being fractures [1]. The burden of MSI is expected to become more significant in coming years with an ageing population and an increase in road traffic accidents in low and middle-income countries, largely due to the increased use of motorised transport and less developed trauma care systems [2]. Considering the probable increase in MSI, the provision of services by healthcare systems to improve health outcomes is crucial. Therefore, an understanding of what facilitates the use of healthcare services, and what influences individuals with MSI to behave differently in relation to their health is urgently needed.

Past studies have demonstrated that the decision to seek healthcare services is influenced by a number of factors including the individual’s health status, socio-demographic characteristics of the individual and their ability to access the type of resources they may need [3–5]. Social support has been identified as a potential factor that may either facilitate (i.e. increase uptake) or buffer (i.e. provide direct support) the uptake of healthcare services [6, 7]. Social support is defined as information leading individuals to believe they are cared for and loved, esteemed and valued, and belong to a network of communication and mutual obligation [8].

The evidence for the relationship between social support and healthcare service use among persons with MSI is limited. Much of what is currently known about social support and healthcare service use has been gathered from research conducted within the general population, older persons, and those with mental illness in which injury effects may be obscured [7, 9–15]. Among these populations, studies have shown mixed evidence for the role of social support on healthcare service use. A study suggests that social support is relatively unimportant when it comes to healthcare service use [9], whereas, other evidence suggests that social support can either enhance or reduce reliance on healthcare services [7, 10–15]. Several studies have also shown that the combination of stressful life events and social support has a modifying effect on healthcare service use [6, 16]; however, these results have not been replicated in all studies [12]. Variations in study populations, social support measures, availability of healthcare resources, and statistical analysis most likely account for these mixed results [7, 9–15]. Another possible reason is that the studies were not designed to explain how social support affects healthcare services utilisation. Thus, the mechanism through which social support influences healthcare service use remains unclear.

In this study, we adopt the Berecki-Gisolf et al. Healthcare Services Utilisation Framework [17] to explore the mechanisms through which social support influences healthcare service use in the MSI population. Berecki-Gisolf et al. proposed an adapted Andersen and Newman Framework of Healthcare Services Utilisation for a compensated population. In a compensated population, financial barriers to healthcare services are removed, under clauses set by the compensation system. In addition to the three factors proposed in the Andersen and Newman framework to explain healthcare service use: 1) predisposition factors (i.e. socio-cultural characteristics) 2) enabling factors (i.e. individual and community factors) and 3) need factors (i.e. health problems) [3], this extended framework proposes the additional following four factors to reflect the compensable context: 1) compensation system (i.e. scheme policies) 2) regulator (i.e. administrating body) 3) provider incentives and 4) individual incentives.

**Fig. 1:**
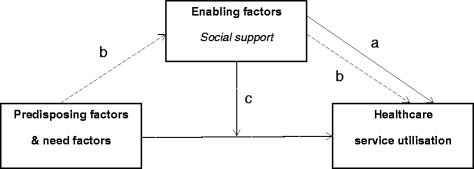
Research model. **a** Direct effect: the link between social support and healthcare service utilisation is significant even after the influence of other predictors variables is taken into account; **b** Mediation: the link between predisposing factors, need factors and healthcare service utilisation operates partly via the effect of social support on healthcare service utilisation; **c** Effect modification: Different level of social support has an intensifying effect on the link between predisposing factors, need factors and healthcare service utilisation

Figure 1 depicts the proposed conceptual framework. Based on this framework, the aims of the study are to investigate 1) the association between social support and healthcare service use (direct effect); 2) whether social support mediates the association between predisposing factors, need factors and healthcare service use (mediator); and 3) whether social support modifies the association between predisposing factors, need factors and healthcare service use (effect modifier).

## Methods

### Study design

We undertook a secondary data analysis of cross-sectional surveys conducted among compensated transport accident victims in Victoria in 2010 and 2011, linked to compensation claims and payment records.

### Ethical considerations

The Monash University Human Research Ethics granted exemption from ethical review as the study satisfies 5.1.22 of the National Statement on Ethical Conduct in Human Research ([18], p.40): “Institutions may choose to exempt from ethical review research that: is negligible risk research; and involves the use of existing collections of data or records that contain only non-identifiable data about human beings”.

### Transport injury compensation system

In the state of Victoria, Australia, those injured in land-based transport accidents involving a car, motorcycle, tram, bus or train are eligible to claim compensation for treatment, income replacement, rehabilitation and long-term support services via the Transport Accident Commission (TAC), regardless of fault. In addition, the TAC provides compensation for injury and death for individuals travelling in a Victorian-registered motor vehicle in other Australian states. Injuries and death occurring on the road but not involving a motorised vehicle (e.g. a collision between a pedal cyclist and a pedestrian) are not eligible for compensation [19].

### Data sources

Data were collected from the Client Outcomes Survey (COS). The TAC conducts an annual COS to measure the health and vocational status of its clients. The survey is designed to inform the TAC about the impact of its claims management practices and the design of the compensation scheme on the health and vocational outcomes of its clients. The survey includes standardised measures of vocational and health status prior to injury, current vocational status, current health status, including physical and mental health, pain, mobility and functional independence, access to and satisfaction with healthcare and satisfaction with the TAC. Data are collected via computer automated telephone interview (CATI) conducted by a third-party social research organisation. The questionnaire takes approximately 25 min to administer.

Data were also collected from the Compensation Research Database (CRD). The CRD is an administrative database held by the Institute for Safety, Compensation and Recovery Research (ISCRR) at Monash University. The database contains de-identified transport-related injury claims data from 1 January 1987 through to 31 December 2014. The database contains one record for every claim received by the TAC, and each record contains information necessary for the management of the compensation claim, including accident, demographic, and injury details, and payments for health and other services. For the purposes of this study, the CRD was linked to the COS via a unique claim identifier.

### Study participants

In 2010 and 2011, a total of 2476 participants completed the COS, including 1649 (67 %) participants with MSI. The sample age ranged from 16–89 years. The sample included active and inactive claims. Active claims were defined as having a payment from the TAC within the last six months prior to being surveyed. Inactive claims were defined as having no payments in the last six months but at least one payment made within seven to 24 months prior to being surveyed. The sample comprised of minor to moderate injuries such as soft tissue or complex orthopaedic/multi-trauma, including mild and moderate brain injury. Catastrophic injuries such as spinal cord injury, severe traumatic brain injury, amputees and burns were excluded. In this study, the sample was limited to participants with MSI including sprains/strains, soft tissues, fractures and dislocations.

### Measures

#### Predisposing characteristics

Predisposing characteristics included gender (female vs. male), age, country of birth (Australia vs. others), education (university level vs. less than university level), employment status at time of accident (yes vs. no), occupation, and socio-economic indexes for areas (SEIFA). Age was defined as the age of claimant at the time of the interview and was categorised into six groups: 16–24, 25–34, 35–44, 45–54, 55–64, and 65+ years. Among those working at the time of the accident, occupation was categorised into eight groups according to the Australian and New Zealand Standard Classification of Occupations (ANZSCO) [20]: managers, professionals, technicians and trade workers, community/personal service workers, clerical/administration workers, sales workers, machine operators/drivers and labourers. SEIFA is a measure of relative disadvantage and advantage based on a range of attributes such as a person’s residential location and income [21]. The distribution of scores was divided into ten equal deciles. A high decile reflects relative advantage. The deciles were recoded into two categories, where decile 1–5 reflected relative disadvantage and deciles 6–10 reflected relative advantage.

#### Need factors

Need factors included physical health, mental health, pre-injury health status (excellent, very good, good, fair and poor), injury types (soft tissue, sprains and strains, fractures, whiplash), time since injury, and hospitalisation (>1 day hospital stay vs. not hospitalised) which was used as a proxy for injury severity [22, 23]. Physical health and mental health were assessed by the Short-Form-12 Health Survey Version 2 (SF-12V2). The SF-12V2 is a validated international tool that consists of twelve questions [24]. The SF-12V2 measures eight concepts: physical functioning, role limitations due to physical health problems, bodily pain, general health, vitality (energy/fatigue), social functioning, role limitations due to emotional problems, and mental health (psychological distress and psychological well-being). Two summary scores were derived: the Physical Component Summary (PCS) and the Mental Component Summary (MCS). The PCS focuses mainly on limitations in physical functioning, role limitations due to physical health problems, bodily pain, and general health. The MCS focuses mainly on role limitations due to mental and emotional problems and social functioning. The scores were derived using Australian weights based on the Australian population norms [25]. Higher scores on the PCS and MCS indicated more positive physical and mental health. Time since injury was derived from the date of the interview and the accident date.

#### Enabling factors

Structure of social support included marital status and number of dependent children. Marital status was grouped into married/de facto relationship, widowed/separated/divorced and never married. Preliminary analysis found an association between marital status and number of dependent children; thus a family structure composite was created. The family composition was categorised into six groups: married/de facto relationship with children, married/de facto relationship with no children, widowed/separated/divorced with children, widowed/separated/divorced with no children, never married with children, never married with no children. Sources and functions of social support included accessing help from family and friends. For family and friends items, participants rated their level of agreement with the following question; *‘Can you get help from family members/friends if you need it?’* on a 4-point scale that ranged from 1 “*yes, definitely”* to 4 “*no, not at all”*.

#### Healthcare service use

Two categories of healthcare services were examined in the two year follow-up period from the date of the accident: allied and mental healthcare services. Allied healthcare services included services provided by physiotherapists, chiropractors, osteopaths, acupuncturists and occupational therapists. Mental healthcare service included services provided by psychiatrists, psychologists, general practitioners (restricted to mental health treatment plan only), social workers and vocational counsellors. Allied healthcare service use was measured as the number of services accessed in the two year follow-up period. Due to the small number of mental health care services accessed, mental health care services use was transformed into a binary variable, those who accessed mental health care services (yes) and those who did not (no).

### Statistical analyses

Descriptive statistics and frequency distributions of key variables are presented. For the direct effect analyses, two types of models were conducted to examine an association between each source of social support and healthcare service use. Allied healthcare services use was analysed using zero-inflated negative binomial regression (ZINB) modelling. ZINB is a maximum-likelihood count regression analysis, designed for non-normal (i.e. skewed and over dispersed) count data with an excess of zero values [26]. The ZINB models the probability of being a non-user versus a user of healthcare services (i.e. the logistic model component) and weighs cases accordingly in order to determine the prediction of healthcare services use intensity (i.e. the negative binomial regression model component). Vuong tests were conducted to assess the appropriateness of a ZINB model against the standard negative binomial regression model. Mental healthcare services use was analysed using logistic regression modelling. Both models were adjusted for predisposing factors and need factors.

For the mediation analyses, we used the Karlson, Holme and Breen (KHB) method [27] to assess whether social support mediates the association between predisposing factors, need factors and each type of healthcare services use. This method provides unbiased decompositions of total effects into direct and indirect effects for both linear and nonlinear models. The decomposition is accomplished by comparing the estimated coefficients obtained from a reduced model (without mediator) to a full model (with mediator). The differences between these two sets of estimated coefficients provide an estimate of the indirect effect (i.e. the part of the total effect running through the mediating variable). However, the KHB method is currently not suitable for count models. Therefore, allied health care service use was transformed from a count variable into a binary variable - those who access allied health care services (yes) and those who did not (no).

Lastly, for the effect modification analyses, we tested interaction effects to see whether social support modifies the association between predisposing factors, need factors and healthcare service use. A ZINB model with interaction effect was used to analyse allied healthcare services use and a logistic regression model with interaction effect was used to analyse mental healthcare services use.

In all statistical models, the *“not often”* category in the sources of social support variables was used as the reference group instead of the *“no, not at all”* category as participants who rated not receiving any support may not be a homogenous group (e.g. participants who did not require any help, or did not have family living in the area). A p-value of less than 0.05 was considered significant in all analyses. Data analyses were conducted using STATA version 12 and SAS version 9.4.

## Results

### Participant characteristics

The characteristics of the study population are presented in Table 1. The mean age of the cohort was 44 years (standard deviation 15) and 59 % of the participants were male. Over half of the participants were married or in a de facto relationship (54 %) and 56 % had children. Three quarters of the participants did not have a university level education (76 %). Three quarters of the participants were born in Australia (75 %). The majority were employed at the time of the accident (80 %). Sixty-one percent of the participants were in the State’s upper 50 % of relative socio-economic advantage, based on their area of residence and income. The most common occupations were technicians and trade workers (22 %), followed by professionals (18 %) and community/personal service workers (13 %). Over half were hospitalised (67 %) after the transport accident, and 57 % sustained fractures. Forty-three percent of the participants rated their health as excellent prior to the accident. The mean PCS and MCS scores of participants were 43.6 and 42.2, respectively. One thousand eighty-three participants (66 %) had a total of 53,687 allied healthcare encounters. In contrast, 453 (28 %) participants accessed a total of 5,463 mental healthcare services. The median numbers and interquartile ranges (IQR) of allied and mental healthcare visits were 31 (13–59) and 6 (3–14), respectively.

**Table 1:** Demographic characteristics of the sample

	N (column %) (*n* = 1649)
Gender	
Male	965 (58.5 %)
Female	684 (41.5 %)
Age group^a^	
16–24	176 (10.7 %)
25–34	307 (18.6 %)
35–44	365 (22.1 %)
45–54	392 (23.8 %)
55–64	247 (15.0 %)
65+	149 (9.0 %)
Marital status^a^	
Married or in de facto relationship	896 (54.3 %)
Widowed/Separated/Divorced	284 (17.2 %)
Never married	459 (27.8 %)
Children^a^	
Yes	918 (55.7 %)
No	717 (43.5 %)
Family composition^a^	
Married or in de facto relationship with children	511 (31.0 %)
Married or in de facto with no children	382 (23.2 %)
Widowed/separated/divorced with children	129 (7.8 %)
Widowed/separated/divorced with no children	154 (9.3 %)
Never married with children	276 (16.7 %)
Never married with no children	176 (10.7 %)
Educational level^a^	
University level education	373 (22.6 %)
Less than University level education	1252 (75.9 %)
Country of birth^a^	
Australia	1243 (75.4 %)
Others	397 (24.1 %)
SEIFA^a^	
Upper 50 % (relative advantage)	1005 (60.9 %)
Lower 50 % (relative disadvantage)	631 (38.3 %)
Employed at the time of accident^a^	
Yes	1320 (80.0 %)
No	325 (19.7 %)
Occupation^a,b^	
Managers	136 (10.3 %)
Professionals	233 (17.7 %)
Technicians and trade workers	293 (22.2 %)
Community/personal service workers	166 (12.6 %)
Clerical/administration workers	132 (10.0 %)
Sales workers	95 (7.2 %)
Machine operators/drivers	100 (7.6 %)
Labourers	158 (12.0 %)
Injury types	
Dislocation	119 (7.2 %)
Fracture	932 (56.5 %)
Soft tissue	517 (31.4 %)
Sprain/strain	81 (4.9 %)
PCS score (mean and sd)	43.6 (7.2)
MCS score (mean and sd)	42.2 (9.8)
Hospitalisation (within 7 days of accident)	
Yes	953 (57.8 %)
No	696 (42.2 %)
Health prior to accident ^a^	
Excellent	704 (42.7 %)
Very good	643 (39.0 %)
Good	241 (14.6 %)
Fair	46 (2.8 %)
Poor	13 (0.8 %)
Time post-injury	
0–12 months	362 (22.0 %)
13–24 months	565 (34.3 %)
25–36 months	379 (23.0 %)
37+ months	343 (20.8 %)
Family support^a^	
Definitely	1087 (66.2 %)
Yes, sometimes	307 (18.7 %)
Not often	98 (6.0 %)
No, not at all	150 (9.1 %)
Friends’ support^a^	
Definitely	942 (57.7 %)
Yes, sometimes	444 (27.2 %)
Not often	112 (6.9 %)
No, not at all	135 (8.3 %)

^a^Data missing ranging from 0.1 to 1.5 %

^b^Restricted to those who were employed at the time of the accident

### Direct effects

Table 2 reports the results of the ZINB regression models, which include a logistic model and a negative binomial model. All ZINB models were adjusted for family composition, predisposing factors (gender, age, country of birth, education and SEIFA) and need factors (physical health, pre-injury health status, injury types, time since injury and hospitalisation). Vuong tests showed the ZINB model as preferred against the standard negative binomial regression models for both family (*z* = 15.12, *p* < 0.01) and friends’ (*z* = 15.12, *p* < 0.01) support.

**Table 2:** Direct effect: Zero inflated negative binomial regressions for family and friends’ support on allied healthcare service use

	Allied healthcare service use			
	Logistic		Negative binomial	
Models	OR	95 % CI	IRR	95 % CI
1. Family^a^				
Definitely	2.17*	1.21–3.91	0.82	0.63–1.06
Yes, sometimes	1.67	0.89–3.14	0.98	0.74–1.30
No, not at all	2.66*	1.34–5.27	0.73	0.53–1.01
Not often (ref)				
2. Friends^a^				
Definitely	1.87*	1.09–3.21	0.65*	0.52–0.83
Yes, sometimes	1.55	0.88–2.72	0.79	0.62–1.01
No, not at all	2.31*	1.20–4.42	0.75	0.55–1.02
Not often (ref)				

*OR* odds ratio; *IRR* incidence rate ratio; *CI* confidence intervals; *REF* reference

In the logistic model, an OR value greater than 1 indicates increasing odds of being a more frequent non-user of healthcare services, whereas an OR value less than 1 indicates increasing odds of being a more frequent users of healthcare services. In the negative binomial model, an IRR value greater than 1 indicates increase healthcare service use rate, whereas an IRR less than 1 indicates decrease healthcare service use rate

^a^Models adjusted for family composition, gender, age, education, country of birth, SEIFA, injury types, pre-injury health status, hospitalisation, days post-injury, and PCS score

*p < 0.05

In the logistic model component of the ZINB model, participants with ‘definite’ (odds ratio (OR), 2.17; 95 % CI, 1.21–3.91) or ‘no’ (OR 2.66; 95 % CI 1.34–5.27) support from family were two and three times as likely to be non-users of allied healthcare services, compared to those with ‘not often’ support from family. In the negative binomial model component of the ZINB model, no statistically significant association was found between family support and allied healthcare service use intensity. In contrast, support from friends was significantly associated with both the use and intensity of allied healthcare services. That is, participants reporting “definite” (OR 1.87; 95 % CI 1.09–3.21) or “no” (OR 2.31; 95 % CI 1.20–4.42) support from friends were twice as likely to be non-users of allied healthcare services, relative to those with “not often” support from friends. Participants with “definite” support from friends had 35 % lower rate of allied healthcare visits (incidence rate ratio (IRR) 0.65; 95 % CI 0.52–0.83), compared to those with “not often” support from friends.

Table 3 presents the results of the logistic regression analyses examining the relationship between social support and mental healthcare service use. All logistic regression models were also adjusted for family composition, predisposing factors (gender, age, country of birth, education and SEIFA) and need factors (mental health, pre-injury health status, injury types, time since injury and hospitalisation). No statistically significant associations were observed with family support and mental healthcare service use. In contrast, the odds of accessing mental healthcare services was 40 % (OR 0.60; 95 % CI 0.38–0.95) lower for participants reporting “definite” support from friends, compared to those reporting “not often” support from friends.

**Table 3:** Direct effect: Logistic regressions for family and friends’ support on mental healthcare service use

	Mental healthcare service use	
Models	OR	95 % CI
1. Family^a^		
Definitely	0.74	0.46–1.21
Yes, sometimes	1.21	0.71–2.04
No, not at all	0.72	0.39–1.34
Not often (ref)		
2. Friends^a^		
Definitely	0.60*	0.38–0.95
Yes, sometimes	0.89	0.55–1.42
No, not at all	0.95	0.54–1.68
Not often (ref)		

*OR* odds ratio; *CI* confidence intervals; * REF* reference

An OR value greater than 1 indicates increasing odds of accessing healthcare services, whereas an OR value less than 1 indicates decreasing odds of accessing healthcare services

^a^All logistic regression models adjusted for family composition, gender, age, education, country of birth, SEIFA, injury types, pre-injury health status, hospitalisation, days post-injury, and MCS score

*p < 0.05

### Mediation

Social support was examined as a mediator of the relationship between predisposing factors, need factors, and healthcare service use. Contrary to the hypothesis, there were no significant mediation effects of family or friends’ support on the uptake of allied and mental healthcare services.

### Effect modification

**Fig. 2:**
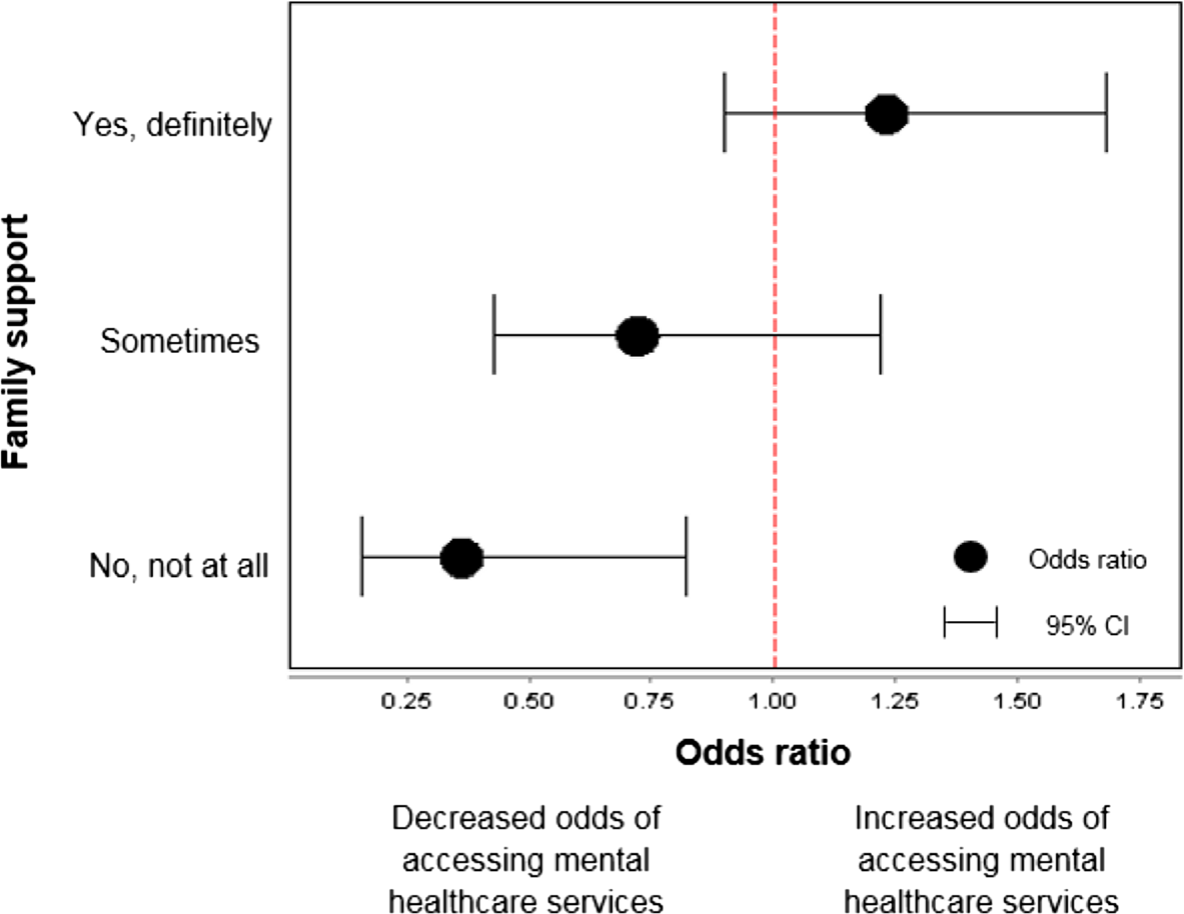
Effect modification: Interaction effect of family support and SEIFA on mental healthcare service use. Comparison of participants in the upper 50 % (relative advantage) to participants in the lower 50 % (relative disadvantage) for various levels of family support. Reference group is “not often support”. An OR value greater than 1 indicates increasing odds of accessing mental healthcare services, whereas an OR value less than 1 indicates decreasing odds of accessing mental healthcare services. Logistic regression model adjusted for family composition, gender, age, education, country of birth, SEIFA, injury types, pre-injury health status, hospitalisation, days post-injury, and MCS score

Potential interaction effects between predisposing factors, need factors and the source of social support on healthcare service use were tested. Family support modified the association between SEIFA and mental healthcare service use. Figure 2 compares participants in the upper 50 % (relative advantage) to participants in the lower 50 % (relative disadvantage) for various levels of family support. The Y axis represents the level of family support and the X axis represents the OR for the relative advantaged group to the relative disadvantaged group. Among those with no social support, the odds of using mental healthcare services in the advantaged group was 64 % lower (OR 0.36; 95 % CI 0.16–0.83) than the odds of using mental healthcare services in the disadvantaged group. There were no significant interaction effects for friends’ support, predisposing factors and need factors on mental healthcare service use.

## Discussion

The aim of this study was to address a gap in the literature through exploring the association between social support and healthcare services utilisation among people with compensable MSI. Understanding the mechanisms through which social support influences healthcare services utilisation can lead to the development of social support interventions and ultimately improve health outcomes. Using Berecki-Gisolf et al. adapted Andersen and Newman Framework of Healthcare Services Utilisation for a compensated population [17], the study explored whether social support (i) had a direct effect on healthcare service use, (ii) mediated the association between predisposing factors, need factors and healthcare service use, and (iii) interacted with various predisposing factors, need factors on healthcare service use. Finally, this paper examined whether these associations varied depended upon the source of support and the type of healthcare services involved. In support of the hypotheses, the findings indicated that social support was a direct factor related to healthcare services, and also an effect modifier, but contrary to the hypothesis, not a mediator*.* The findings suggest that the role of social support is complex and appears to vary depending on the source of support and the type of healthcare services accessed.

The results potentially suggested that individuals with greater social support make less use of healthcare services than to those with less support. This finding is inconsistent with past research conducted within the general population [7], older persons [13] and mental illness [14, 15] populations which found that individuals with greater social support were more likely to seek general medical services. We found that greater support from family and friends were associated with lower uptake of allied healthcare services. Greater support from friends was also associated with lower rate of allied healthcare services. Family relationships are the earliest and often the most enduring of social ties. Families also tend to provide a substantial amount of support in many areas of daily life following injury [28]. Similarly, friendships tend to grow stronger over long periods of time and are linked to positive physical and mental health outcomes [29]. Thereby, supportive relationships may reduce allied healthcare service use by providing direct support. Alternatively, the results may suggest that supportive relationships substitute for formal treatment or perhaps even delay help seeking behaviour.

Interestingly, we also found that no support from family and friends was associated with lower uptake of allied healthcare services. This is inconsistent with the literature which suggests that when support from family and friends are limited, people are more likely to access healthcare services provided by healthcare professionals [6, 11]. In our study, the absence of social support may have decreased access to healthcare services because there was a lack of health knowledge sharing among social network members and no provision of help from the social network to enable health care seeking behaviour. Alternatively, individuals with no support may have had difficulties in navigating the compensation system in order to access healthcare services. In a compensation system, requests for compensable treatment are mostly required to be approved by the insurer prior to commencement of the service. A qualitative study conducted by Murgatroyd et al. [30] showed that delayed treatment approvals and lack of consistent decision making between insurer and healthcare professionals resulted in dissatisfied participants. In our study, among those with no social support, 30 % had lower satisfaction with the TAC (data not shown). Those with no social support and lower satisfaction with the TAC were also more likely to have a high SEIFA. Given the timeliness of and access to healthcare services via the compensation system, it is possible that these results suggest that individuals with no support and high SEIFA sought healthcare services outside of the compensation system as there were reduced financial barriers. Further research is warranted to explain the relationship between lack of support and healthcare service use.

Furthermore, we found that greater support from friends, but not from family, were associated with lower uptake of mental healthcare services. The results suggest that support from friends reduces the need for mental healthcare services through providing another avenue for support. Research has shown that friends are associated with numerous mental health benefits [29]. For example, having a friend to confide in provides emotional support and contributes to a sense of belonging and overall well-being. This is consistent with past studies conducted in the general population which found that individuals with greater social support were less likely to seek mental healthcare services [7, 10, 12]. In contrast, we found no effect of family support on mental healthcare service use. A likely explanation for the differential effect is that individuals had a greater tendency to confide in friends than family. Past research suggests that family relationships tend to be more complex and viewed by individuals as ambivalent, that is, both closer and more troublesome than friendships [31].

We found that social support from family and friends did not mediate the relationship between predisposing factors, need factors and allied and mental healthcare services. This finding indicates that predisposing factors and need factors do not facilitate the use of healthcare services via social support. There is sparse research in this area, with the majority of research focusing on the role of social support in mediating stress and health outcomes [8, 32, 33]. Further research is needed to understand the precise nature of the relationship between predisposing factors, need factors, social support, and healthcare service use.

For mental healthcare service use, significant interaction effects emerged between family support and SEIFA. Individuals with no family support and a lower SEIFA were associated with a higher likelihood of consulting a mental health professional than those with a higher SEIFA. This finding suggests that in a no-fault compensable population, financial barriers to healthcare services are reduced, thereby allowing individuals with easy access to healthcare services regardless of socio-economic status and family support. This is inconsistent with past research examining solely SEIFA and healthcare service use. Some studies found that the uptake rates for psychological services decreased among people from more disadvantaged areas, compared to those from less disadvantaged areas [34, 35]. In contrast, a study conducted by Dal Grande et al. reported that the proportion of people who accessed psychiatry and psychological services did not vary by SEIFA [36]. However, these studies did not explicitly test for interaction effects of social support and SEIFA on the uptake of mental healthcare services. Further research is warranted to support the relationship between SEIFA, social support and mental healthcare service use.

Although this study provides greater insight into the role of social support and healthcare service use, several limitations of this study must be noted. By using an existing survey generated by the TAC, the study was restricted in its measures of social support. First, the social support measure was non-standardised and it is therefore not known if the items measured were what they were intended to measure. Second, the social support item was based on a single dimension of social support. There are several dimensions to social support including informational, tangible, appraisal and emotional support; thus, it is plausible that the type of support affects the use of healthcare services differently. Third, only the perception of social support was assessed. It is possible that the size of the participants’ social network, the pattern of interaction within the social networks, and the content of advice given by the social network members influences healthcare service use. Future research should investigate supportive exchanges between individuals and social network members in order to better understand how and when social network members either prevent or encourage the uptake of healthcare services. Fourth, we were unable to examine whether social support had an impact on healthcare service use outside of the compensation system, that is, services paid for by a private insurer, or services fully billed to Medicare, the Australian universal healthcare program [37]. Fifth, we recognised that the decision to engage with healthcare services is influenced by a variety of psychosocial and socio-economic factors including social support. Future research is required to determine which factors have the greatest impact on the use of healthcare services. Finally, the sample included in this study should not be considered to be representative of the population of individuals with MSI. All participants were injured in a transport accident within a jurisdiction that provides no-fault compensation for healthcare services. This relatively unique situation reduces financial barriers in accessing healthcare services. It also provides data that allows detailed examination of healthcare service use. The study is therefore unlikely to represent those cases of MSI without access to a similar compensation system (e.g. those incurring a MSI via sport, recreation or home). Furthermore, the extent to which these results are applicable to other jurisdictions will need to be considered given the various transport accident compensation schemes in Australia (i.e. hybrid, fault-based, no-fault). Future research is needed to determine the extent to which these findings can be generalised to other jurisdictions, non-compensable transport injury, and to injury that is not transport-related.

There are several implications arising from this study for the prevention, treatment and intervention of individuals with MSI. Regarding prevention, the findings suggest that individuals can cope better with MSI by drawing on the strength of their social support network and decreasing the need for healthcare services. Social support networks should therefore be protected, strengthened and mobilised as potential channels to provide health education and information to alleviate distress. Regarding treatment, these findings suggest that healthcare service providers could potentially engage appropriate individuals within one’s social network (e.g. spouse/friends) in the health treatment plan. Prior to the development of a health treatment plan, healthcare service providers could assess the abilities, assets and capacities of an individual’s social network including potential challenges in the required uptake of healthcare services. Regarding intervention, interventions could be developed and delivered to sustain support services, particularly for those with limited social support. In addition, healthcare service providers could direct intervention efforts toward helping individuals to develop skills that are needed to mobilise and maintain the individual’s existing social support network. They may also aid in the development of new networks such as patient support groups to promote optimal health service use for those without access to a social support network.

## Conclusions

In summary, the findings of the current study highlight the importance of social support in accessing healthcare services following a MSI sustained in a transport accident. Although the results varied across sources of support and different types of services, the findings suggest that social support has a direct and modifying effect on healthcare service use. The findings do not suggest that social support mediates the association between predisposing factors, need factors and healthcare service use. This study contributes to the existing literature through clarifying the mechanisms of social support in healthcare service utilisation. The study findings have implications for the role of social support in the treatment and intervention of individuals with MSI.

## Abbreviations

ANZSCO, Australian and New Zealand Standard Classification of Occupations; CATI, computer automated telephone interview; COS, client outcomes survey; CRD, Compensation Research Database; IQR, interquartile ranges; IRR, incidence rate ratio; ISCRR, Institute for Safety, Compensation and Recovery Research; KHB, Karlson, Holme and Breen; MCS, mental component summary; MSI, musculoskeletal injuries; OR, odds ratio; PCS, physical component summary; SEIFA, socio-economic indexes for areas; SF-12V2, Short-Form-12 Health Survey Version 2; TAC, Transport Accident Commission; ZINB, zero-inflated negative binomial regression
